# Predicting Delay in Goal-Directed Action: An Experience Sampling Approach Uncovering Within-Person Determinants Involved in the Onset of Academic Procrastination Behavior

**DOI:** 10.3389/fpsyg.2021.695927

**Published:** 2021-07-22

**Authors:** Lena M. Wieland, Ulrich W. Ebner-Priemer, Matthias F. Limberger, Ulrike E. Nett

**Affiliations:** ^1^Chair of Applied Psychology, Mental mHealth Lab, Institute of Sports and Sports Science, House of Competence, Karlsruhe Institute of Technology, Karlsruhe, Germany; ^2^Empirical Educational Research, University of Augsburg, Augsburg, Germany

**Keywords:** delay behavior, procrastination, self-regulation, intention-action gap, experience sampling

## Abstract

Academic procrastination involves the delayed implementation of actions required to fulfill study-related tasks. These behavioral delays are thought to result from momentary failures in self-regulation (i.e., within-person processes). Most previous studies focused on the role of trait-based individual differences in students’ procrastination tendencies. Little is known about the within-person processes involved in the occurrence of procrastination behavior in real-life academic situations. The present study applied an event-based experience sampling approach to investigate whether the onset of task-specific delay behavior can be attributed to unfavorable changes in students’ momentary appraisals of tasks (value, aversiveness, effort, expectations of success), which may indicate failures in self-regulation arise between critical phases of goal-directed action. University students (*N* = 75) used an electronic diary over eight days to indicate their next days’ intentions to work on academic tasks and their task-specific appraisals (*n* = 582 academic tasks planned). For each task, a second query requested the next day determined whether students’ task-related appraisals changed and whether they implemented their intention on time or delayed working on the respective task (*n* = 501 completed task-specific measurements). Students’ general procrastination tendency was assessed at baseline using two established self-report questionnaires. Stepwise two-level logistic regression analyses revealed that within-person changes in task-related appraisals that reflected a devaluation of the study-related tasks increased the risk for an actual delay. The risk to delay decreased when students maintained a positive attitude toward the task. Students’ general procrastination tendency did not predict individual differences in their task-specific delay behavior. We discuss these findings in light of the growing effort to understand the within-person processes that contribute to induce procrastination behavior under real-life academic conditions and illustrate how this knowledge can benefit the design of tasks and instructions that support students’ self-regulation to their best.

## Introduction

Delaying work on a task involves the intention to perform a goal-directed action but to postpone its implementation until a later time (Lay, 1986; Steel et al., 2001). This delay causes an intention-action gap, the core criterion for procrastination, which is further characterized by the awareness that the delay is to one’s own disadvantage (Steel, 2007; Simpson and Pychyl, 2009; Klingsieck, 2013). These disadvantages become most evident in academic settings where definite deadlines limit the time available to accomplish study-related tasks. There is ample evidence for a negative relationship between the pronounced tendency to delay study-related tasks (i.e., academic procrastination) and students’ academic performance (Tice and Baumeister, 1997; Steel et al., 2001; van Eerde, 2003; Richardson et al., 2012). In addition, increased procrastination tendencies were found to be positively related to indicators of impaired mental and physical well-being (e.g., Tice and Baumeister, 1997; Sirois et al., 2003; Grunschel et al., 2013; Krause and Freund, 2014; Beutel et al., 2016). These findings become even more concerning given that many students (30 to 45% of respondents) have been found to procrastinate on study-related tasks (e.g., writing term papers or studying for exams) frequently and view their behavior as problematic (Solomon and Rothblum, 1984; Beswick et al., 1988; Day et al., 2000; Schouwenburg, 2004).

To elucidate why many students engage in such an evidently dysfunctional behavior, research has typically focused on relating between-person differences in students’ general procrastination tendencies to a set of characteristic trait patterns (for overviews, see Ferrari et al., 1995; van Eerde, 2003; Steel, 2007; Klingsieck, 2013). At the same time, a growing body of research has suggested that students’ procrastination behavior (i.e., actual delays in working on tasks) results from more temporary failures in self-regulation (e.g., Steel et al., 2001; Dewitte and Schouwenburg, 2002; Howell et al., 2006; Howell and Buro, 2009; Sirois and Pychyl, 2013). Effective self-regulation would require that individuals apply regulatory strategies that allow them to adapt their cognition, motivation, their affective responses, and their behavior to deal successfully with a given task (e.g., Winne and Hadwin, 1998; Boekaerts, 1999; Zimmerman, 2002; Pintrich, 2004; Efklides, 2011). Thus, to understand procrastination behavior as a consequence of self-regulatory failure, it would be appropriate to consider both trait-based individual differences and more situation-, task-, or context-dependent determinants that change within the individual over time (i.e., within-person processes). One explanatory approach that highlights this requirement is the mood-repair hypothesis proposed by Sirois and Pychyl (2013). This approach builds upon the transactional stress model of Lazarus and Folkman (1984) and explains procrastination behavior as a maladaptive coping strategy that serves to avoid an unpleasant affective state that arises when the demands of a task seem to exceed one’s abilities, competencies or available resources. Cross-sectional designs and self-report questionnaires – assessing individual differences in students’ general procrastination tendency – preclude the possibility of recognizing the within-person processes or context-specific influences involved in the occurrence of delay behavior under real-life conditions (see also van Eerde, 2003; Molenaar, 2004; Schmitz, 2006).

The few studies that have used behavioral measures to examinestudents’ delay behavior over time, and under real-life conditions, have revealed that students’ task-specific delay behavior was subject to time-dependent fluctuations in general (e.g., Steel et al., 2001; Moon and Illingworth, 2005; Howell et al., 2006; Krause and Freund, 2014), and discontinuously declined over time toward the deadline (as proposed by Temporal Motivation Theory, Steel and König, 2006; Steel et al., 2018). Other studies used experience-sampling approaches to show that an increased occurrence of procrastination behavior was related to everyday stresses (such as negative affect, Pollack and Herres, 2020; or poor sleep quality, van Eerde and Venus, 2018), providing additional support for the theoretical propositions of the mood-repair hypothesis (Sirois and Pychyl, 2013). However, these studies are still in the minority. The potential impact of task- or context-dependent variability in behavioral determinants (i.e., within-person variability) on students’ actual behavior (and on the occurrence of behavioral delays) has rarely been studied (van Eerde, 2003; Voelkle et al., 2014), although there has been an encouraging increase in studies that have made an effort to address this research gap over the past ten to twenty years (e.g., Pychyl et al., 2000; Krause and Freund, 2014; Steel et al., 2018; van Eerde and Venus, 2018; Pollack and Herres, 2020; Svartdal et al., 2020). The present study sought to complement previous research on potential indicators for self-regulatory failures that are thought to precede the occurrence of task-specific delay behavior under real-life conditions. The study goes beyond the analysis of between-person differences to examine whether changes in behavioral determinants that arise in the course of action within individuals and may indicate a failure of self-regulation can predict the actual occurrence of task-specific behavioral delays.

## From Between- to Within-Person Perspectives in Research on Procrastination

A large body of previous research on procrastination has been based on the assumption that individuals possess a more or less pronounced procrastination tendency (Ferrari, 1991; Schouwenburg and Lay, 1995; van Eerde, 2003; Schouwenburg, 2004). Numerous studies have examined between-person differences in students’ self-reported procrastination tendencies using procrastination scales or inventories (for reviews, see van Eerde, 2003; Steel, 2007; Klingsieck, 2013). These studies demonstrate associations between self-reported procrastination tendencies and certain personality traits (a lack of conscientiousness, elevated levels of neuroticism, or impulsivity), some have even described procrastination as a trait-like construct in itself (see Johnson and Bloom, 1995; Watson, 2001; van Eerde, 2003; Schouwenburg, 2004; Steel, 2007).

More comprehensive explanations suggest that procrastination results from self-regulatory failure, as the individual fails to direct one’s cognition, motivation, and behavior to the attainment of some long-term goal (e.g., Dewitte and Lens, 2000; Wolters, 2003; Howell et al., 2006; Steel and König, 2006; Sirois and Pychyl, 2013). Studies following this rationale have provided evidence that pronounced procrastination tendencies are related to unfavorable motivational beliefs or attitudes. Students who are primarily motivated by extrinsic rewards (Senécal et al., 1995; Brownlow and Reasinger, 2000), hold mastery-avoidance or work-avoidance orientations (Wolters, 2003; Howell and Watson, 2007; Howell and Buro, 2009), or report a lack of self-efficacy for self-regulation (Klassen et al., 2008), were frequently found to report pronounced procrastination tendencies. Moreover, students with pronounced procrastination tendencies appear to use hardly any (meta-)cognitive strategies when working on academic tasks (Wolters, 2003; Howell and Watson, 2007; Corkin et al., 2011), which makes it difficult to regulate their behavior effectively. The relevance of intra-individual processes of self-regulation in the occurrence of procrastination behavior is most explicitly stated in the mood-repair hypothesis presented by Sirois and Pychyl (2013). This proposition has been supported by empirical findings linking students’ procrastination tendencies to their experience of negative emotions or their inability to regulate these emotions adequately (e.g., Lay, 1992; Tice et al., 2001; McCown et al., 2012; Rebetez et al., 2015; Eckert et al., 2016; Pollack and Herres, 2020).

While postulating that self-regulatory failures (i.e., within-person processes) determine the occurrence of procrastination behavior, most previous studies have related individual differences in students’ procrastination tendencies to individual differences in determinants deemed relevant for self-regulation (i.e., general interests, abilities, or attitudes). However, the success or failure of self-regulation does not depend on students’ trait-like characteristics, abilities, or attitudes alone. Instead, self-regulatory processes mediate the complex interplay between trait-like determinants (including abilities and attitudes), contextual or situational influences (e.g., task characteristics or affective states), and students’ actual learning behavior or performance (e.g., Winne and Hadwin, 1998; Boekaerts, 1999; Zimmerman, 2002; Pintrich, 2004; Efklides, 2011). Thus, to understand behavioral delays as a result of self-regulatory failure, it will be indispensable to consider behavioral determinants that may change dynamically over time within individuals, depending on task- or context-specific influences. Specifically, this would require to capture the occurrence of a delay, that is, the absence of an intended action (Lay, 1986; Svartdal et al., 2018), and to examine whether within-person changes in behavioral determinants contribute to the occurrence of this delay.

## The Onset of Delays in Goal-Directed Action

### Any Delay Requires an Intention

At the beginning of every self-regulated action, an individual has to form the intention to strive for a goal, to reach a certain condition or performance standard (Heckhausen and Gollwitzer, 1987; Austin and Vancouver, 1996; Pintrich, 2004). The actual translation of this intention into goal-directed action will be crucially influenced by its strength (i.e., its temporal stability), which is itself determined by subjective cost-benefit considerations (Gollwitzer, 1990; Ajzen, 1991; Sheeran and Abraham, 2003; Cooke and Sheeran, 2004; Steel and König, 2006). The costs and benefits of pursuing one goal must be weighed against those of pursuing various other alternatives. Two key determinants are relevant for these considerations: the expectation that one will be able to perform the behavior that leads to the desired outcome successfully and the subjective value attached to that outcome (Atkinson, 1957; Gollwitzer, 1990; Bandura, 1997; Eccles and Wigfield, 2002; Locke and Latham, 2002; Steel and Weinhardt, 2018). The higher the subjective value of the anticipated outcome and the expectation that goal-directed behavior can be successfully implemented, the higher the willingness of the person to invest effort and to translate an intention into action (Brehm and Self, 1989; Gollwitzer, 1990; Klein et al., 1999; Eccles and Wigfield, 2002; Dietrich et al., 2017).

Modern expectancy-value theory (e.g., Eccles and Wigfield, 1995; Wigfield and Eccles, 2000; Eccles, 2005) conceptually separated the expectancy determinant into more domain-specific ability beliefs and task-specific expectations of success. However, students’ ability beliefs and expectations of success have been found to be highly correlated in real-life academic settings (Eccles and Wigfield, 2002; Dietrich et al., 2017). Since the present study was designed to examine students’ task-specific delay behavior, we will focus on students’ task-specific *expectations of success* throughout the following. Moreover, the value determinant has been separated into four conceptual sub-components: attainment value, intrinsic value, utility value, and costs (Eccles and Wigfield, 2002; Eccles, 2005); all but the latter have been found to be highly correlated within an academic domain or learning situation (e.g., Trautwein et al., 2012; Dietrich et al., 2017). For the present study, we focus on the attainment value sub-component, which reflects the personal importance of successful task accomplishment (e.g., Wigfield and Cambria, 2010). However, the costs associated with a task (e.g., the perception of how much effort is required for successful task accomplishment) can be distinguished empirically from the remaining value components (e.g., Trautwein et al., 2012; Flake et al., 2015; Dietrich et al., 2017). Therefore, we follow Barron and Hulleman’s (2015) suggestion and consider students’ appraisal of task-specific effort costs (the term *effort* is used throughout the following) as a third determinant of their behavioral intentions. Another determinant that has been shown to increase the risk that an intention will not be realized in time is the individual’s perceived aversion toward engaging in a task (e.g., Lay, 1992; Milgram et al., 1995; Blunt and Pychyl, 2000). While task aversiveness is a multifaceted construct (for a detailed analysis, see Blunt and Pychyl, 2000), most findings suggest that tasks perceived as aversive seem to be less personally meaningful and generally affectively unpleasant (Lay, 1992; Milgram et al., 1995; Blunt and Pychyl, 2000). Therefore, it seems highly likely that perceptions of task aversiveness will affect one’s commitment to engage in goal-directed action (see also Blunt and Pychyl, 2000). Task aversiveness was thus included as the fourth relevant determinant of students’ willingness to engage with their tasks in the present study.

However, the mere formation of a strong intention does not guarantee task accomplishment (Heckhausen and Gollwitzer, 1987; Gollwitzer, 1990; Ajzen, 1991; Cooke and Sheeran, 2004). The number of intentions to work on academic tasks expressed by students with pronounced procrastination tendencies is comparable to that of other students, but they are significantly more likely to delay their realization (Steel et al., 2001; Dewitte and Schouwenburg, 2002). Therefore, the delay cannot result alone from a lack of initial willingness. Instead, meta-analytical evidence suggests that it is the temporal stability of intentions that moderates their predictive value for the performance of corresponding behavior (Cooke and Sheeran, 2004).

### Any Delay Is the Deviation From an Intention

The model of action phases (Heckhausen and Gollwitzer, 1987; Gollwitzer, 1990) describes a temporal sequence of different stages that have to be passed during goal-directed action. After intention formation (predecisional phase), volitional action stages involve the planning of specific strategies (preactional phase), which must then be translated into goal-directed action (actional phase) in order to realize the intention (Heckhausen and Gollwitzer, 1987; Gollwitzer, 1990). Various difficulties can arise both within and in the transition between these phases, posing a challenge for self-regulation (discussed in detail by Gollwitzer and Sheeran, 2006; Wieber and Gollwitzer, 2010). Self-regulation theories (e.g., Winne and Hadwin, 1998; Boekaerts, 1999; Zimmerman, 2002; Pintrich, 2004; Efklides, 2011) have focused precisely on those dynamic adaptions that support the realization of task-specific behavioral intentions. Especially in the face of difficulties, distractions, or attractive alternative options to satisfy one’s needs, it may become necessary to increase one’s (self-regulatory) efforts to adhere to the original intention (see Gollwitzer, 1990; Sheeran et al., 2005). Under such circumstances, the person must ascertain whether the additional effort required to realize the intention is as yet justified.

Effective self-regulation would involve intraindividual processes that constantly (re)assesses whether an intended action (e.g., working at a task) should be initiated, maintained, changed, or terminated under the given circumstances (e.g., Pintrich, 2000; Zimmerman, 2002; Inzlicht et al., 2014). Moreover, the (cognitive, affective, motivational) capacities of the individual stand in a reciprocal relationship to situational or contextual influences, and it is this reciprocal relationship that ultimately affects the behavior (e.g., Kuhl, 1992; Winne and Hadwin, 1998; Boekaerts, 1999; Pintrich, 2004; Sirois and Pychyl, 2013). Therefore, an individual’s decision to delay or work on a specific task should not be influenced only by the intention that was based on the outcome of previous cost-benefit considerations. Instead, the willingness to engage in goal-directed action may change depending on the current circumstances.

Some studies have recently revealed that motivational determinants related to students’ performance behavior are not merely a stable characteristic of the individual, but also significantly influenced by situation and task characteristics (e.g., Vancouver and Kendall, 2006; Tanaka and Murayama, 2014; Martin et al., 2015; Dietrich et al., 2017). Most notably, a significant amount of variance in the determinants of students’ goal-directed actions was within-person variance at the (domain, day, or) task level (e.g., Vancouver and Kendall, 2006; Tanaka and Murayama, 2014; Dietrich et al., 2017). Moreover, affective experiences have been identified as one of the major determinants for the occurrence of procrastination behavior (Sirois and Pychyl, 2013). Procrastination was found to be particularly likely to occur for tasks that students perceived as being particularly aversive, unpleasant, difficult, boring, or effortful (Lay, 1992; Blunt and Pychyl, 2000; Ferrari and Scher, 2000; Pychyl et al., 2000), and was related to everyday stresses (such as negative affect, Pollack and Herres, 2020; or poor sleep quality, van Eerde and Venus, 2018), providing additional support for the claim that the occurrence of procrastination behavior is not only determined by individual trait-based influences, but also affected by rather situational or context-specific influences. Further research focusing on within-person processes is necessary to gain a more comprehensive insight into the relationship between self-regulatory failures and the occurrence of procrastination behavior under real-life academic conditions. It is needed to extend research that focused on individual differences in procrastination tendencies to the momentary, task- and situation-specific changes in behavioral determinants that occur within individuals over time to obtain a more complete picture of the conditions that increase students’ risk to delay working on their academic tasks.

## The Present Study

The primary objective of the present study was to investigate whether the occurrence of behavioral delays would be predicted by within-person changes in students’ cognitive-affective appraisals of tasks that arise between different phases of goal-directed action. We further sought to examine whether within-person changes in the appraisal of tasks have an effect on the occurrence of task-specific delay behavior that goes beyond the influence of between-person differences in general procrastination tendencies.

While between-person differences in procrastination tendencies were assessed using established self-report questionnaires, an event-based experience sampling approach was implemented (a) to identify within-person changes in students’ cognitive-affective appraisals of tasks, and (b) to capture the momentary occurrence of task-specific delay behavior in their everyday life. For one week, students’ intentions to work on academic tasks and their initial task-specific appraisals were captured each evening using electronic diaries (e-diaries). For each task, a second assessment was requested the next day to determine whether students realized the intention or delayed working on the respective task.

We expected that students’ initial appraisal of a task (i.e., the expectation of success, task value, anticipated effort, and task aversiveness) in the early phase of planning (i.e., during intention formation) would predict the occurrence of task-specific delay behavior (*Hypothesis 1*). We further account for the fact that these appraisals may change between the phase of intention formation and the moment that the intention should be actually realized by goal-directed action. It was expected that the risk to delay a task should (a) decrease as the perceived task value increases between the intention formation and the moment that the intention should be realized, but (b) increase as the subjective aversiveness of the task or the anticipated effort increase between the intention formation and the moment that the intention should be realized (*Hypothesis 2*). These within-person changes in students’ cognitive-affective appraisals of tasks were expected to be strong indicators for the occurrence of task-specific delay behavior, in addition to effects that were expected by individual differences in general procrastination tendencies (*Hypothesis 3*).

## Materials and Methods

### Participants and Procedure

Participants were recruited within cross-curricular courses that were offered for all students enrolled at a large German University (with technical focus) to foster students’ self-regulation and time-management skills. The study was conducted in two waves because of limited course capacity, including *n* = 29 students from a course provided during winter term and *n* = 46 students from two courses provided during the summer term. The overall sample comprised *N* = 75 students (*M*_*age*_ = 23.07, *SD*_*age*_ = 2.28, *n* = 74) of diverse majors^1^ (*n* = 43 Bachelor; *n* = 31 Master). Demographic information was missing for one participant, five participants did not indicate their gender (*n* = 50 male).

The compact cross-curricular courses started during the third week of lectures during winter and summer term, respectively. Students were informed about the study in the first session and were introduced to the handling of the e-diary that was preinstalled on smartphones with Android systems (Movisens GmbH, 2015/2016). Students who agreed to participate, and gave their informed consent, filled out paper-pencil questionnaires to gather demographic information and to assess their procrastination tendency at baseline. Finally, participants received a smartphone with the e-diary.

On Sunday evening after the introductory session, an audible signal emitted by the smartphones reminded participants that they were supposed to respond to the first e-diary query. That query was the starting signal for the following eight days of experience sampling beginning on Monday. The second session of the cross-curricular courses was scheduled for the week after the eight days of experience sampling (nine days including the starting signal). Course content regarding self-regulation and time-management strategies that might affect participants’ behavior was not provided before the second session.^2^ Following local legislation and institutional requirements, ethical review and approval were not required for the present study. However, all procedural steps of the study were reviewed for compliance with local data protection laws and followed international ethical standards (American Educational Research Association, 2011). Participants were rewarded for their participation with additional course credit. Cinema vouchers (5.0 € value) were provided as an incentive for students with an overall compliance of at least 80% completed e-diary queries.

### Experience Sampling Procedure

Participants’ delay behavior was captured using an event-based experience sampling approach that allowed for the observation of delays in realizing intended goal-directed actions at the moment of their occurrence. The outcome of interest was the event when a participant decided to realize an intention (i.e., working on the task), or to delay the realization of that intention (i.e., not working on the task at the intended time). Therefore, the e-diary was programmed to cover two separate assessment units for each task. Planning task-specific intentions (T0 measurement) was triggered by fixed-time prompts every evening (between 8:30 pm and 9:00 pm). Participants were initially asked to indicate at least two tasks (e.g., ‘study for exam’ or ‘exercise’) that they intended to work on the next day. It was not specified that these had to be academic tasks, but it was stated in the introductory session that academic tasks were of primary interest to our research. Whenever participants missed a fixed-time prompt, they could press a button appearing on the screen between 9:00 pm. and 11:00 pm. to elicit the planning-phase themselves. When planning a task, participants were further asked to indicate the intended time (hh:mm) for working on their task the following day. The specified time defined the moment that the intention was to be realized by taking goal-directed action and triggered the second unit of assessment (T1 measurement).

Both units of assessment encompassed questions regarding participant’s subjective appraisals of the tasks. Planning task-specific intentions (T0 measurements) included the appraisal of the subjective task value, students’ task-specific expectation of success, task aversiveness, and the anticipated effort required to work on the task. At the moment that the intention was to be realized (T1 measurements), each prompt was followed by displaying the planned task on the screen; students were then again requested to provide their momentary task-specific appraisals (on the subjective task value, task aversiveness, and anticipated effort required). Hereafter, participants were asked to indicate whether they follow their intention and work on the task or delay working on that task.^3^

The first T0 measurement was triggered on Sunday evening (after the introductory session), so that participants could plan their tasks for Monday. The last day of experience sampling (Monday one week later) included T1 measurements for the tasks planned the previous day, but did not include another T0 measurement. Therefore, students used the e-diary for nine days, but task-specific assessments were requested for a total of eight days only, since each task-specific assessment included two measurements, the first in the evening (T0) and the second the following day (T1).

**FIGURE 1:**
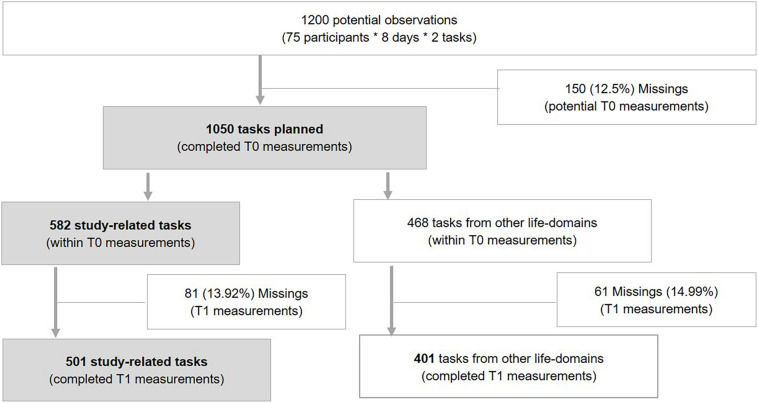
Data flow indicating the subset of Level 1 observations available for the analyses conducted to answer the research questions under investigation. In total, *n* = 501 Level 1 observations fulfilled the eligibility criteria (shadowed boxes): participants (Level 2; *N* = 75) planned a task (T0 measurements); indicated that the task was study-related, and completed the intention-realization assessments (T1 measurements) for these tasks.

Over the eight days of experience sampling (nine days including theinitial T0 assessment), the *N* = 75 participants (Level 2) planned *n* = 1050 tasks (Level 1) out of 1200 tasks thatcould have potentially been planned (seeFigure 1 for a detailed flowchart). Bothassessment units (T0 and T1 measurements) were completed for a total of *n* = 908 tasks. Therefore, the average compliance rate (completed task-specific measurements) was 86.48%, based on *n* = 1050 tasks planned. As our research question focused on delays in working on academic tasks, only those measurements that were indicated as being study-related were used in the analyses. As such, the final subset of observations (Level 1) included *n* = 501 academic tasks (see Figure 1). In 78.8% of the cases (T1 measurements), participants indicated that they worked on their study-related task (*n* = 501) at the time intended, whereas 21.2% of the tasks were indicated as being delayed.^4^ Thus, according to the results of a simulation study by Schoeneberger (2016), our sample (*N* = 75 participants at Level 2 and *n* = 501 task-specific measurements at Level 1) meets the requirements to achieve sufficient power to detect the expected effects in logistic multi-level models (described in more detail in the data analysis section).

### Measures

#### Delay Behavior

During each intention-formation (T0 measurement), participants were asked to indicate a “goal or task” that they intended “to work on the following day” within a short text field. Task-specific delay behavior was measured during the intention-realization measurement (T1) by asking participants whether they will “begin to work on the task or goal right now” (the respective task was presented on the screen). The response scale for this item was binary, with a *yes* response (coded 0) indicating that the participant followed the intention to work on the task, whereas a *no* response (coded 1) indicated behavioral delay.

#### Momentary Task-Specific Appraisals

Single items were used to assess students’ momentary task-specific appraisals within both task-specific measurements (T0 and T1). The application of single-item measures can be justified for experience sampling studies to minimize participant burden, increase participants’ willingness to respond accurately, and prevent increased drop-out rates (e.g., Gogol et al., 2014). It has also been demonstrated that single-item measures can have favorable psychometric properties under certain conditions (e.g., Robins et al., 2001; Hoeppner et al., 2011; Lucas and Donnellan, 2012; Goetz et al., 2016).

The items used to assess students’ task-specific appraisals were held virtually parallel in wording between the first (T0) and the second measurement (T1). The only adjustment was that items presented during T0 measurements referred to the task planned for *tomorrow*, whereas items presented during T1 measurements referred to the task that the participant intended to work on *right now*. Each item was answered on a visual analog scale, ranging from 0 to 100, with verbal anchors adjusted to the appraisal requested (for descriptive statistics of the single-item measures, see Table 1). All the items presented in the e-diary were presented in German language and were only translated into English for this publication (the German wording of the items can be found in the Appendix).

**TABLE 1:** Descriptive statistics (grand mean, SD, range), ICC and level-specific bivariate correlations for the unstandardized task-specific appraisals (Level 1; *n* = 501 study-related tasks) indicated by the participants (Level 2; *N* = 75) during planning (T0) and intention realization assessments (T1).

	*M*	*SD*	*Min; Max*	ICC	1	2	3	4	5	6	7
1 Value (T0)	74.93	17.01	6.0; 100.0	0.40	−	0.53**	0.03	0.06	0.11	0.12*	0.43***
2 Value (T1)	72.70	18.89	2.0; 100.0	0.43	0.88***	−	−0.04	−0.09	0.12*	0.09	0.42***
3 Avers (T0)	59.19	17.50	0.0; 100.0	0.37	−0.19	−0.19	−	0.41***	0.35***	0.32***	−0.18**
4 Avers (T1)	59.20	18.33	0.0; 100.0	0.43	−0.11	−0.20	0.98***	−	0.20**	0.41***	−0.14*
5 Effort (T0)	66.56	18.56	7.0; 100.0	0.32	0.41**	0.37*	0.28	0.30	−	0.56***	−0.12*
6 Effort (T1)	65.28	18.02	6.0; 100.0	0.21	0.35*	0.22	0.45**	0.43**	0.97***	−	−0.04
7 Expect (T0)	70.36	18.74	2.0; 100.0	0.30	0.41*	0.33*	−0.58***	−0.52***	0.18	0.09	−

Correlations above the diagonal indicate correlations at the within-person level (Level 1); correlations below the diagonal indicate correlations at the between-person level (Level 2). ICC, intraclass correlation coefficient; Value, task value; Avers, task aversiveness; Effort, effort required for working on the task; Expect, expectation of success; T0, first task-specific measurement during intention formation (planning); T1, second task-specific measurement at the time intended for working on a task (intention realization). *p < 0.05, **p < 0.01, ***p < 0.001.

The subjective *value* of the task was assessed by askingparticipants, “How important is it to you personally that you workon that task/reach that goal [right now/tomorrow]” – (*notimportant at all* to *very important*). The *expectation* of success was assessed (exclusively during T0 measurements) by the item: “How likely do you think it is that you will work on that task/reach that goal tomorrow” – (*very unlikely* to *very likely*). These items were adapted from previous studies (Oettingen et al., 2015; Kappes and Oettingen, 2014; Sevincer et al., 2014). We further assessed the anticipated *effort* required for working on a task as the third behavioral determinant (e.g., Eccles, 2005; Barron and Hulleman, 2015) by the item: “[Prospectively,] How much effort do you have to invest [right now] to work on this task/reach this goal?” – (*very little* to *very much*). While task aversiveness is probably a more multifaceted construct (cf. Blunt and Pychyl, 2000), a person’s aversion about a task has often been assessed by asking whether a task is perceived as more or less “pleasant” or “unpleasant” in previous work (Solomon and Rothblum, 1984; Lay, 1990; Blunt and Pychyl, 2000). Therefore, we decided to capture participants’ subjective appraisal of the pleasantness (vs. aversiveness) of a task by the item: “How (un-)pleasant is this task/working on this goal [right now]” – (*very unpleasant* to *very pleasant*). Task aversiveness ratings have been reverse coded for the analyses so that higher values indicate that a task was perceived as more aversive (less pleasant).

#### Procrastination Tendencies

Students’ procrastination tendencies were assessed at baseline, using the German version of the Tuckman Procrastination Scale (TPS-d: Stöber, 1995; TPS, Tuckman, 1991) as a more general measure of (trait-like) procrastination tendencies, and the German version of the Academic Procrastination State Inventory (APSI-d: Helmke and Schrader, 2000) as a more proximal measure for students’ (state-like) academic procrastination tendencies.

The Tuckman Procrastination Scale (TPS in the following) consists of 16 items describing behaviors or attributions that indicate a tendency to delay the start or completion of tasks or goal-directed actions in general (e.g., “When I have a deadline, I wait till the last minute” Tuckman, 1991, p. 477). Answers were provided on a five-point Likert-type scale, ranging from *this is not at all true* (1) to *this is very true* (5). Participants (*N* = 74, information missing for one participant) reached an average sum score of 56.87 (*SD* = 8.68; Range = 32.00–75.00) in the present study, Cronbach’s alpha was 0.83 within our sample.

The 12-item *state-procrastination* subscale of the German version of the Academic Procrastination State Inventory (APSI-d: Helmke and Schrader, 2000; originally developed by Schouwenburg, 1995) asks for the frequency of interruptions or distractions that occurred during learning activities *within the lask week*. Therefore, the APSI-d assesses procrastination tendencies in a more time- and context-specific way. In the present study, the sample (*N* = 74, information missing for one participant) reached a mean score of 1.89 (*SD* = 0.63; Range: 0.42–3.08) for the *state-procrastination* subscale (hereafter APSI-p). Cronbach’s alpha was 0.79 within our sample.

### Data Analysis

We accounted for the nested data structure of task-specific measurements (Level 1, *n* = 501) within participants (Level 2, *N* = 75) in the analyses using Mplus (Mplus Version 8.1; Muthén and Muthén, 1998–2018). We were primarily interested in predicting events of delay based on students’ task-specific expectations of success (assessed during intention formation, T0), and on within-person changes in their subjective appraisals (task value, task aversiveness, and required effort) between intention formation (T0) and intention realization (T1) measurements. Predictor variables were prepared by initially z-standardizing all T0 measurements (see Table 1 for descriptive statistics of the unstandardized variables). These z-standardized T0 measurements were decomposed into their between-level (Level 2, person mean) and within-level (Level 1, person-mean centered) components.

To examine whether delays in the realization of intentions to work on study-related tasks can be predicted by within-person changes in task-specific appraisals (at Level 1), indicators quantifying these changes were needed. Therefore, assessments of task value, aversiveness, and effort measured at T1 were standardized using the grand-mean and standard deviation of the T0 measurements before subtracting the standardized T0 measurements from these standardized T1 measurements. In doing so, we receive a variable that represents changes in task value, task aversiveness, and effort evaluations between the task-specific measurements (changes from T0 to T1). These indicators were not centered at the person-mean to facilitate the interpretation of their effects by keeping a meaningful zero point (see Enders and Tofighi, 2007), which indicates that the appraisal of a task did not change between the two measurements. Finally, the TPS (trait procrastination) and the APSI-p (state procrastination) score was calculated for each participant to quantify individual differences in procrastination tendencies at baseline. The resulting variables were z-standardized and used as between-level predictors in the logistic two-level regression analyses.

A stepwise approach was used to predict the risk for the occurrence of task-specific delays, considering the impact of multiple predictors in eight logistic multilevel regression models. The outcome variable of interest is the binary indicator for whether a student reported to work on a task (*Y* = 0) or to delay working on that task (*Y* = 1). All models were computed using full information maximum likelihood estimation (MLR, maximum likelihood estimation with robust standard errors), random-intercepts^5^, but fixed effects for predictor variables at the level of task-specific measurements (Level 1). The null model (intercept-only model) was computed to predict the average risk (logit of odds) for delays when none of the assessed predictors was included. To test our first Hypothesis, four logistic two-level regression models were analyzed (Model 1 through Model 4), including each of the task-specific appraisal dimensions (task value, task aversiveness, effort, and expectation of success) separately. To test the effects of the initial task-specific appraisals, the within-level components of T0 measurements were entered as predictors at Level 1. To test the effects of within-person changes in task-specific appraisals (task value, task, aversiveness, and effort), change indicators were entered as predictors at Level 1. Task-specific expectations of success were measured at T0 exclusively, so that there was only one predictor variable (i.e., the person-mean centered T0 assessment) included at Level 1 (i.e., Model 4). Finally, the person-mean (across tasks) of each predictor variable was included as a Level 2 covariate in each model to control for differences in students’ average appraisals of their study-related tasks (e.g., some students may consistently score higher in their appraisal of task value than others). Two additional models were analyzed to examine the effects of between-person differences (at the level of students, Level 2) in baseline measures of trait-procrastination (TPS, Model 5) and state-procrastination (APSI-p, Model 6) on the risk that students delayed (vs. worked on) their tasks.

Our second hypothesis was tested in a combined analysis (Model 7), including all predictor variables reflecting students’ subjective appraisals of tasks. Finally, to test our third hypothesis, we added the baseline measures of trait-procrastination (TPS) and state-procrastination (APSI-p) as predictors to the between-person level (Level 2) of the combined model (Model 8). This final step in the analysis was necessary to determine whether the predictive influence of task-specific appraisals and momentary changes in these appraisals (i.e., the within-person effects of task-specific determinants) on the risk that a task was delayed (vs. worked on) would persist when accounting for individual differences in general procrastination tendencies. The model fit for each model was compared against the null model – Model 8 was compared against Model 7 – using chi-square difference tests based on log-likelihood values and scaling correction (Satorra and Bentler, 2010; Muthén and Muthén, 2018).

## Results

### Descriptives

There was no significant difference in general procrastinationtendencies [TPS, *t*(72) = 0.21, *p* = 0.83; APSI-p, *t*(72) = 0.31; *p* = 0.76] betweenstudents that participated during winter or summer term (Winter: *M*_*TPS*_ = 56.41, *SD*_*TPS*_ = 8.57; *M*_*APSI–p*_ = 1.92, *SD*_*APSI–p*_ = 0.57; Summer: *M*_*TPS*_ = 57.16, *SD*_*TPS*_ = 8.83; *M*_*APSI–p*_ = 1.88, *SD*_*APSI–p*_ = 0.67).

On average, each student completed both task-specific measurements for 6.68 tasks using the e-diary. Table 1 provides descriptive statistics for the task-specific assessments (task value, task aversiveness, effort, and expectation of success) before standardization or person-mean centering. Table 2 provides descriptive statistics for the standardized between-level components (person-means at Level 2) and the within-level components (person-mean centered at Level 1) of each task-specific appraisal dimension (task value, task aversiveness, effort, and expectations of success) that was assessed during intention-formation (T0). Table 2 also shows descriptive statistics for the variables that indicate within-person changes in the appraisals between T0 and T1 assessments (i.e., changes in task value, task aversiveness, and effort). Moreover, Table 2 provides information on the units of increase that will support the interpretation of effects in the logistic two-level regression analyses.

**TABLE 2:** Descriptive statistics for standardized variables (subjective task-specific appraisals) used as predictors in the logistic two-level regression analyses at Level 1 (task-specific ratings; *n* = 501) and Level 2 (person-mean ratings; *N* = 75 participants).

	Level 2			Level 1			
	*M*	*SD*	*Min; Max*	*M*	*SD*	*Min; Max*	units of increase^*a*^
Value (T0)	0.00	0.70	−2.24; 1.47	0.00	0.71	−3.24; 2.38	0.59
Value (T1–T0)	−	−	−	−0.13	0.86	−4.17; 4.35	0.59
Avers (T0)	0.00	0.67	−1.75; 2.05	0.00	0.75	−2.25; 2.32	0.57
Avers (T1–T0)	−	−	−	0.00	0.89	−5.25; 2.51	0.57
Effort (T0)	0.00	0.65	−1.72; 1.71	0.00	0.76	−3.50; 2.32	0.54
Effort (T1–T0)	−	−	−	−0.07	0.81	−3.23; 3.66	0.54
Expect (T0)	0.00	0.64	−1.79; 1.51	0.00	0.77	−2.42; 2.85	0.53

*Task-specific ratings have been divided by 10 before standardizing. Level 2: person-mean values of standardized T0 ratings; Level 1: person-mean centered values of standardized T0 ratings; time-dependent differences (change) in task-specific assessments (T1–T0) at Level 1 were not centered at the person mean. Value, task value; Avers, task aversiveness; Effort, effort required; Expect, expectation of success; T0, first task-specific measurement; T1, second task-specific measurement; T1–T0, change in task-specific ratings between measurements. ^a^Value that equals to an increase of 10 points on the original scale.*

With no predictor variables entered to the logistic two-level regression model (null model), the threshold risk for task-specific delays was *B* = 2.093 (*p* < 0.001). There was significant between-person variance in tasks being delayed vs. worked on (*s*^2^ = 3.217; *p* = 0.001; 95% CI = 1.368; 5.066), indicating that 49% of the relative risk to delay (vs. work on) academic tasks was explained by between-person variance in students’ delay patterns (ICC = 0.49)^6^.

### Predicting Behavioral Delay by Within-Person Change Mechanisms

Results of the first four models (Model 1 – Model 4) computed to determine the effects of within-person variability in task-specific appraisals (initial assessment and change indicator at Level 1) – controlling for differences in students’ average appraisals of their study-related tasks (person-mean across tasks at Level 2) – on the relative risk that a task is being delayed (vs. worked on) are depicted in Table 3. Each of these models had a significantly better fit than the null model (Table 4 provides model fit information).

**TABLE 3:** Distinct multi-level-models predicting the risk to delay (*Y* = 1) vs. work on a task (*Y* = 0), based on initial task-specific assessments (T0) and change indicators (T1–T0).

		*B* (*SE*)	*p*	95% CI	OR	*R*^2^ (*p*)
**Model 1.**						
Threshold		**2.278** (0.331)	<0.001	1.630; 2.926	−	
L1	Val_*T*__1__–__*T*__0_	−**0.951** (0.197)	<0.001	−1.338; −0.564	0.386	**0.173** (0.007)
L1	Val_*T*__0__/pmc_	−**0.866** (0.297)	0.003	−1.447; −0.285	0.412	
L2	Val_*T*__0__/pm_	−0.542 (0.333)	0.103	−1.195; 0.111	−	0.051 (0.441)

**Model 2.**						
Threshold		**2.225** (0.357)	<0.001	1.525; 2.962	−	
L1	Ave_*T*__1__–__*T*__0_	**0.749** (0.263)	0.004	0.234; 1.264	2.115	0.103 (0.114)
L1	Ave_*T*__0__/pmc_	**0.691** (0.291)	0.018	0.120; 1.261	1.995	
L2	Ave_*T*__0__/pm_	**1.086** (0.386)	0.005	0.329; 1.843	−	0.178 (0.091)

**Model 3.**						
Threshold		**2.089** (0.339)	<0.001	1.425; 2.753	−	
L1	Eff_*T*__1__–__*T*__0_	0.201 (0.273)	0.461	−0.334; 0.737	1.223	**0.020** (0.419)
L1	Eff_*T*__0__/pmc_	0.361 (0.214)	0.092	−0.059; 0.781	1.435	
L2	Eff_*T*__0__/pm_	0.075 (0.425)	0.860	−0.757; 0.907	−	0.001 (0.929)

**Model 4.**						
Threshold		**2.313** (0.367)	<0.001	1.594; 3.032	−	
L1	Exp_*T*__0__/pmc_	−**1.013** (0.248)	<0.001	−1.499; −0.527	0.363	**0.156** (0.017)
L2	Exp_*T*__0__/pm_	−**1.238** (0.526)	0.019	−2.269; −0.206	−	0.154 (0.210)

*L1, Level 1 (n = 501 tasks); L2, Level 2 (N = 75 participants); B, regression coefficient(log odds); CI, confidence interval; OR, odds ratio; Threshold, random parameter; Val, task-value; Ave, task aversiveness; Eff, effort required; Exp, expectation of success; T1–T0, change-parameter (difference between task-specific measurements); T0/pmc, within-level parameter for the first measurement (T0) centered at the person mean (pmc); T0/pm, between-level parameter, person mean (pm) of the first measurement **(T0)**. Significant estimates printed in bold.*

**TABLE 4:** Model fit information for the six distinct and two combined logistic multi-level models predicting the risk to delay (*Y* = 1) vs. work on a task (*Y* = 0).

Model	AIC	BIC (n-adjusted)	Chi-square difference test^*a*^
Null Model	452.64	454.73	−
Model 1	421.13	426.34	TRd = 609.49 > *χ*^2^ (3) = 11.35; *p* < 0.001
Model 2	435.57	440.79	TRd = 701.19 > *χ*^2^ (3) = 11.35; *p* < 0.001
Model 3	455.12	460.33	TRd = 625.46 > *χ*^2^ (3) = 11.35; *p* < 0.001
Model 4	412.54	416.71	TRd = 569.50 > *χ*^2^ (2) = 13.82; *p* < 0.001
Model 5	445.33	448.44	TRd = 742.33 > *χ*^2^ (1) = 6.63; *p* < 0.001
Model 6	446.71	449.81	TRd = 960.84 > *χ*^2^ (1) = 6.63; *p* < 0.001
Model 7	400.11	413.66	TRd = 735.00 > *χ*^2^ (11) = 24.73; *p* < 0.001
Model 8	396.61	412.13	TRd = 10.74 > *χ*^2^ (2) = 9.21; *p* < 0.001

*Chi-square difference tests showed the relative superiority to the null model, based on log-likelihood values and scaling correction factors, using Satorra-Bentler test statistic (Satorra and Bentler, 2010; Muthén and Muthén, 2018), Model 8 was tested against Model 7. Two within-level and one between-level predictor for task value (Model 1), task aversiveness (Model 2), and the effort required for working on a task (Model 3); one within-level and one between-level predictor for task-specific expectations of success (Model 4); Models 5 and 6 each included one between-level predictor (TPS; APSI-p). Predictors included in Model 1 through 4 were combined in Model 7; Model 8 included all predictors included in Model 1 through 6. ^a^, followed by “all p < 0.001.”*

Results of Model 1 show that the average risk that a task was delayed (vs. worked on) was *B* = 2.278 (*p* < 0.001) when all predictors covering task value assessments are zero.^7^ The risk that a task was delayed (vs. worked on) decreases significantly with one unit increase in the initial (T0) assessment of task value (*B* = −0.866; *p* = 0.003; OR = 0.41).^8^ Moreover, the risk that a task was delayed (vs. worked on) decreases significantly when the subjective value of the task increases by one unit, from T0 to T1 (*B* = −0.951; *p* < 0.001; OR = 0.39). Between-person differences in the initial task value assessments (person-mean across tasks at Level 2) had no significant effect on the risk that a task was delayed (vs. worked on).

Results of Model 2 show that the average risk that a task was delayed (vs. worked on) was *B* = 2.225 (*p* < 0.001) when all predictors representing task aversiveness are zero. The risk that a task was delayed (vs. worked on) increases significantly when the initial (T0) assessment of task aversiveness (*B* = 0.691; *p* = 0.018; OR = 2.00) increases by one unit. The risk that a task was delayed (vs. worked on) increases significantly when the subjective aversiveness of the task increases by one unit, from T0 to T1 (*B* = 0.749; *p* = 0.004). The relative risk of delaying a task compared to working as intended doubles when task aversiveness increases by one unit between intention formation and intention realization assessments (OR = 2.12). The risk that a task was delayed (vs. worked on) increases significantly for students whose task ambiguity appraisal (across tasks at Level 2) exceeded the sample’s average (*B* = 1.086; *p* = 0.005).

Results of Model 3 show that the average risk that a task was delayed (vs. worked on) was *B* = 2.089 (*p* < 0.001) when all predictors representing the appraisal of effort were zero. Contrary to our expectations, neither the initial appraisal of the effort required for working on a task (T0 assessments) nor the change indicator contributed significantly to the prediction of tasks being delayed (vs. worked on). This also holds for between-person differences in students’ average initial appraisal on the effort required for their tasks (person-mean across tasks at Level 2).

Results of Model 4 show that the average risk that a task was delayed (vs. worked on) was *B* = 2.313 (*p* < 0.001) when students’ prospective expectations of success were zero. As expected, the risk for a task being delayed (vs. worked on) decreases significantly when task-specific expectations of success exceed the person’s mean by one unit (*B* = −1.013; *p* < 0.001). Students with an average expectation of success (person-mean across tasks, Level 2) that exceeds the average of the sample have a significantly lower risk of delaying (vs. working on) their tasks (*B* = −1.238; *p* = 0.019). Results of Model 5 and Model 6 (see Table 5) show that the average risk that tasks were delayed (vs. worked on) was not significantly affected by students’ baseline procrastination tendencies (TPS and APSI-p).

**TABLE 5:** Multi-level-models predicting the risk to delay (*Y* = 1) vs. work on a task (*Y* = 0), based on individual differences in trait- and state-procrastination tendencies.

	*B* (*SE*)	*p*	95% CI	*R*^2^ (*p*)
**Model 5.**				
Threshold	2.148 (0.348)	<0.001	1.466; 2.831	
TPS	0.324 (0.311)	0.299	−0.287; 0.934	0.030 (0.591)
**Model 6.**				
Threshold	2.140 (0.346)	<0.001	1.462; 2.819	
APSIp	−0.026 (0.268)	0.923	−0.550; 0.499	0.000 (0.962)

*Predictors are between-level variables (Level 2), N = 74 participants. B, regression coefficient (log odds); CI, confidence interval; OR, odds ratio; TPS, Tuckman Procrastination Scale (baseline measure); APSIp, Academic Procrastination State Inventory (baseline measure).*

To test our second hypothesis, all predictors were entered into the combined model (Model 7). The combined model had a significantly better fit than the null model (see Table 4). The threshold indicates that the average risk that a task was delayed (vs. worked on) was *B* = 2.486 (*p* < 0.001) when all predictors are zero. Initial task value and task aversiveness appraisals (T0 assessments) lose their predictive power in the combined analysis (see Table 6). However, the risk to delay (vs. work on a task) was affected by students’ task-specific expectations of success (T0 assessments), even in the combined model (see Table 6). Moreover, in accordance with the separate analyses, results of the combined model revealed that the risk to delay (vs. work on a task) was significantly related to task-specific within-person changes in students’ value (*B* = −0.821; *p* < 0.001) and task aversiveness (*B* = 0.588; *p* = 0.021) appraisals. None of the remaining indicators for task-specific appraisals reached significance in this model, which also applies to the Level 2 covariates. Overall, the results of Model 7 revealed that task-specific within-person effects explained 30% of the variance (*R*^2^ = 0.299, *p* < 0.001), whereas between-person differences have not significantly contributed to the explanation of variance (*R*^2^ = 0.243, *p* = 0.081) in students’ task-specific delay behavior.

**TABLE 6:** Combined multi-level-models predicting the risk to delay (*Y* = 1) vs. work on a task (*Y* = 0), based on initial task-specific assessments (T0) and change indicators (T1–T0).

	Model 7^*a*^				Model 8^*b*^	
	*B* (*SE*)	*p*	95% CI	OR	*B* (*SE*)	*p*
Threshold	**2.486** (0.362)	<0.001	1.776; 3.197	−	**2.537** (0.358)	<0.001
**L1.** Val_*T1*__–__*T0*_	−**0.821** (0.212)	<0.001	−1.236; −0.405	0.440	−**0.824** (0.212)	<0.001
**L1.** Ave_*T1*__–__*T0*_	**0.588** (0.254)	0.021	0.089; 1.086	1.800	**0.583** (0.253)	0.021
**L1.** Eff_*T1*__–__*T0*_	−0.060 (0.259)	0.817	−0.567; 0.447	0.942	−0.003 (0.259)	0.990
**L1.** Val_*T0/pmc*_	−0.570 (0.302)	0.059	−1.162; 0.022	0.566	−0.597 (0.307)	0.052
**L1.** Ave_*T0/pmc*_	0.375 (0.262)	0.152	−0.138; 0.888	1.455	0.307 (0.253)	0.226
**L1.** Eff_*T0/pmc*_	0.325 (0.214)	0.128	−0.094; 0.743	1.384	0.339 (0.213)	0.112
**L1.** Exp_*T0/pmc*_	−**0.726** (0.271)	0.007	−1.258; −0.194	0.484	−**0.685** (0.271)	0.011
**L2.** Val_*T0/pm*_	−0.244 (0.398)	0.540	−1.023; 0.536	−	−0.491 (0.379)	0.195
**L2.** Ave_*T0/pm*_	0.880 (0.468)	0.141	−0.229; 1.605	−	0.482 (0.453)	0.288
**L2.** Eff_*T0/pm*_	−0.034 (0.495)	0.945	−1.003; 0.936	−	0.065 (0.484)	0.892
**L2.** Exp_*T0/pm*_	−0.830 (0.605)	0.170	−2.015; 0.355	–	−0.902 (0.617)	0.144
**L2.** TPS_*bl*_	−	−	−	−	0.397 (0.147)	0.274
**L2.** APSIp_*bl*_	−	−	−	−	−0.185 (0.462)	0.252

*B, regression coefficient (log odds); CI, confidence interval; OR, odds ratio; Threshold, random parameter; L1, Level 1; L2, Level 2; Val, task value; Ave, task aversiveness; Eff, effort required for task; Exp, expectation of success; T1–T0, change parameter [difference between second (T1) and first measurement (T0)]; T0/pmc, within-level parameter of the first measurement (T0) centered at the person mean (pmc); T0/pm, between-level parameter, person mean (pm) of the first measurement (T0); TPS_bl_, Tuckman Procrastination Scale; APSIp_bl_, Academic Procrastination State Inventory. Significant estimates printed in bold. ^a^**n**_L1_ = 501 tasks, **R**_L1_^2^**=** 0.299 (p < 0.001); **N**_L2_ = 75 participants, **R**_L2_^2^ = 0.243 (p = 0.081). ^b^**n**_L1_ = 497 tasks, **R**_L1_^2^**=** 0.290 (p < 0.001); **N**_L2_ = 74 participants, **R**_ L2_^2^ = 0.283 (p = 0.054).*

Finally, to test our third hypothesis, the baseline measures for trait-procrastination (TPS) and state-procrastination (APSI-p) were added to the between-person level of the model (Model 8). Model 8 had a significantly better fit than Model 7 (see Table 4). The results obtained from Model 8 show that measures of individual differences in procrastination tendencies (TPS and APSI-p assessed at baseline) do not predict differences in students’ task-specific delay behavior in real-life academic situations (detailed results depicted in Table 6). However, the risk to delay (vs. work on a task) was substantially affected by students’ initial task-specific expectations of success and by within-person changes in their task value and aversiveness appraisals (see Model 8, Table 4).

## Discussion

Although it has been frequently suggested that procrastination results from the failure of self-regulatory mechanisms (e.g., Dewitte and Lens, 2000; Wolters, 2003; Steel and König, 2006; Howell and Watson, 2007; Sirois and Pychyl, 2013), most previous studies neglected that this assumption cannot be comprehensively tested based on the cross-sectional examination of between-person differences. The present study addressed this problem by using an event-based experience-sampling approach to investigate whether the occurrence of task-specific delay behavior can be attributed to failures in self-regulation, which are expressed by unfavorable task-specific appraisal mechanisms, evolving between critical phases of goal-directed action. Overall, our study results show that their tasks’ subjective momentary appraisal predicted student’s dilatory behavior. Moreover, the findings supported our theoretical prediction that within-person changes in the subjective momentary appraisals of study-related tasks evolving between critical stages of goal-directed action predicted the occurrence of dilatory behavior in real-life academic settings. Between-person differences in general procrastination tendencies have not significantly contributed to the prediction of students’ delay behavior patterns.

### Task-Specific Determinants of Delay Behavior: The Initial Appraisal of a Task

In line with our first hypothesis, task-specific within-person differences in students’ expectations of success, task value, and task aversiveness assessed during intention formation predicted the occurrence of delays when the different appraisal dimensions were examined independently. These findings suggest that students tend to delay working on those tasks for which they see lower chances of success, to which they attach lower value (or lower personal importance), and which they perceive as particularly aversive compared to their average task-specific evaluations.

Our results correspond to the findings of previous studies, which indicate that students who have less confidence in their ability to complete academic tasks successfully procrastinate more frequently than those who have stronger competency or self-efficacy beliefs (e.g., Ferrari et al., 1992; Lay, 1992; Wolters, 2003; Wäschle et al., 2014). Moreover, our findings provide further evidence that the expectancy of being able to accomplish the task successfully protects students from delaying goal-directed learning behavior (Wäschle et al., 2014). However, to our knowledge, the present study is the first to demonstrate a direct relationship between student’s task-specific efficacy beliefs (i.e., expectations) and the occurrence of task-specific delay behavior in real-life academic settings.

The influence of the personal value attributed to the achievement of academic tasks has received surprisingly little attention in previous studies on potential determinants of procrastination. This is particularly astonishing because task value is explicitly emphasized in theoretical explanations of the origins of dilatory behavior (e.g., Steel and König, 2006; Glick and Orsillo, 2015; Steel and Weinhardt, 2018). In line with theoretical assumptions, our study revealed that tasks to which students initially attributed an above-average value were significantly less likely to be delayed. This suggests that the occurrence of dilatory behavior might be prevented if students perceive the accomplishment of tasks as personally valuable (or useful; cf. Wäschle et al., 2014). Moreover, in conjunction with the moderately strong, positive correlations between value appraisals and expectations of success (within-level correlations), it seems plausible that the protective effects of above-average ratings on both of these dimensions can be at least partially attributed to the existence of stronger goal commitments (Hollenbeck and Klein, 1987; Gollwitzer, 1993; Klein et al., 1999; Wieber and Gollwitzer, 2010). This is also consistent with findings by Dietrich et al. (2017), indicating that students invested more effort in learning in a given situation if they attached above-average expectations or values to the respective task or topic. In summary, our findings substantiate those of previous studies and indicate that a lack of commitment (or motivation, Locke et al., 1988; Locke and Latham, 1990) increases the risk to delay one’s task-specific action contrary to one’s original intention.

The finding that tasks initially perceived as particularly aversivewere more likely to be delayed is consistent with the results ofprevious studies using diaries (Ferrari and Scher, 2000) or experiencesampling with pagers (Pychyl et al., 2000). Cross-sectional research hasalso revealed that students report procrastinating more frequentlywhen faced with typical academic tasks that are perceived asexceptionally aversive, unpleasant, or unenjoyable (e.g., Milgram et al., 1988, 1995; Lay, 1992). Based on these findings, procrastination behavior has been explained as an impulsive avoidanceresponse to an (affectively) negative experience that occurs whenfacing a task that cannot be aligned with one’s present needs orappears to exceed one’s current resources and abilities(Flett et al., 1995; Blunt and Pychyl, 2000; Tice et al., 2001; Sirois and Pychyl, 2013). This proposition has been further supported by empirical findings that link pronounced procrastination tendencies with an increased experience of or intolerance toward negative emotions and with the inability to adequately regulate these emotions (e.g., McCown et al., 2012; Rebetez et al., 2015; Eckert et al., 2016; Pollack and Herres, 2020). Blunt and Pychyl (2000) have further examined the meaning of students’ task aversiveness perceptions across different stages of goal pursuit. Their findings revealed, among other things, that tasks that were postponed because they were perceived as being aversive were also frequently experienced as being frustrating or boring. According to Pekrun’s control-value theory of achievement emotions (Pekrun, 2006; Pekrun et al., 2007)^9^, feelings of frustration and boredom depend on perceptions of control over the outcome of an achievement-related activity and on the value attached to that outcome. Frustration should arise when a student appraises the outcome of an achievement-related activity as being valuable but has the expectancy of lacking control over achieving this outcome (Pekrun, 2006). Feelings of boredom should arise when students do not ascribe enough value to the outcome of an achievement-related activity, which may be due to a lack of control over the outcome or to task demands falling far below students’ abilities (Pekrun et al., 2007). While we cannot say whether students evaluated a task as being particularly aversive because they anticipated that the task-specific activity might frustrate or bore them, based on the results of the present study. We would like to suggest that future studies might take this possibility into account.

In contrast to previous studies, where students’ preference for avoiding effort was associated with elevated procrastination tendencies (e.g., Ferrari and Scher, 2000; Wolters, 2003; Howell and Watson, 2007), our results did not reveal that the effort expected for performing a task predicted the occurrence of task-specific delay behavior. However, the results show that the effort that was anticipated as being required for task accomplishment was most strongly related to students’ appraisals of task aversiveness (within-person). The present study results likely differ from previous findings because we did not focus on students’ general procrastination tendencies but rather on their self-reported, momentary, and task-specific delay behavior. The effort required to accomplish a task may have an impact on the occurrence of delays only in the long term (in distal goal striving) when the person’s resources are gradually depleted (Baumeister et al., 2000; Muraven and Baumeister, 2000; Inzlicht and Schmeichel, 2012). Thus, our focus on proximal, task-specific behavioral intentions may have led to a situation in which the effort required to accomplish the tasks was rather small. However, students’ ratings for task-specific effort did not differ substantially in range compared to the other appraisal dimensions.

Although not very strong, we did find a positive relationship between task-specific value and effort appraisals at the within-person level. This suggests that students do not necessarily experience task-specific effort costs as being negative. Based on their empirical analysis of different cost components (including the costs associated with the “loss of valued alternatives” and the “outside effort costs” associated with other activities), Flake et al. (2015) argued that considering different cost components is important for improving our understanding of what motivates or constrains students’ engagement in a subject (or task). Thus, it is possible that the task-specific effort costs that have been addressed in the present study do not cover the cost components that are related to the occurrence of task-specific delay behavior. The examination of costs connected with the loss of valued alternatives may be one promising area for research that could contribute significantly to understanding the onset of procrastination behavior.

### Task-Specific Determinants of Delay Behavior: Effects of Within-Person Change

Whereas students’ prospective expectations of success consistently predicted the risk for behavioral delays, initial appraisals of task value and task aversiveness lost their predictive power as soon as the different appraisal dimensions were examined together in a combined multivariate analysis. Instead, and corresponding to our second hypothesis, momentary (time-dependent) within-person changes in students’ task-specific value and aversiveness appraisals predicted the occurrence of dilatory behavior consistently. Specifically, the results of the combined models revealed that the risk to delay the accomplishment of a task decreased when the task’s value increased between the two task-specific measurements. The risk of a delay increased with an increase in the perceived aversiveness between the intention formation and the moment that the intention was to be realized. Overall, these results suggest that behavioral delays were much more likely to occur when students devalued their tasks compared to their initial evaluation. Vice-versa, the risk of delaying goal-directed actions decreased in cases where students succeeded in maintaining a positive attitude toward the task. Thus, if a delay occurred at the time scheduled for realizing their intention, students apparently did not apply effective strategies – including (meta-)cognitive as well as emotion-regulation strategies – to maintain a positive attitude toward their task. Therewith, our findings are in line with the idea that inadequate self-regulation contributed to the occurrence of dilatory behavior (e.g., Dewitte and Schouwenburg, 2002; Steel and König, 2006; Sirois and Pychyl, 2013; Steel et al., 2018).

Previous cross-sectional studies revealed that students who lack abilities to self-regulate their learning behavior are generally more inclined to procrastinate on their study-related tasks (e.g., Wolters, 2003; Howell and Watson, 2007; Klassen et al., 2008; Corkin et al., 2011). However, self-regulated learning is conceptualized as an intra-individual, task- and context-specific process (e.g., Winne and Hadwin, 1998; Zimmerman, 2002; Pintrich, 2004; Inzlicht et al., 2014). These processes cannot be illustrated by cross-sectional sampling plans but should be investigated within more extensive longitudinal research designs (e.g., Schmitz, 2006). With their longitudinal study, Wäschle et al. (2014) provided a good example. Their results show that students use more cognitive strategies to self-regulate their learning and reduce procrastination if they consider the respective learning goal personally valuable (Wäschle et al., 2014). This also supports the interpretation that the present results reflect the proximate intra-individual (time-dependent, within-person) association between self-regulatory failures and the occurrence of task-specific delay behavior in real-life academic settings.

Although our results provide evidence that the occurrence of delay behavior was associated with a momentary devaluation of the task, we cannot draw conclusions about why students’ initial task-specific appraisals have changed. Following the assumptions of Temporal Motivation Theory (TMT; Steel and König, 2006; Steel and Weinhardt, 2018), it is quite possible that the devaluation of a task resulted from a direct comparison with a potentially more attractive alternative activity. However, the present investigation was not supposed to and cannot provide evidence for the temporal discounting principle proposed in TMT (Steel and König, 2006; Ainslie, 2012), as no comparison with an alternative activity was made.

In the present study, task-specific appraisals provided when theintention was formed were used as a reference for the comparison with those provided when the intention was to be realized. Thereby, the present study has demonstrated that intra-individual devaluation processes are involved when students delay the accomplishment of their tasks. These findings are in line with the theory that procrastination behavior is an impulsive avoidance response to (affectively) negative experiences that occur when an individual has to deal with a task (Sirois and Pychyl, 2013). This proposition has been previously supported by studies that related students’ procrastination tendencies to more pronounced experiences of negative emotions or the inability to regulate these emotions adequately (e.g., McCown et al., 2012; Rebetez et al., 2015; Eckert et al., 2016; Pollack and Herres, 2020). However, insight into the dynamic processes that affect procrastination behavior under everyday conditions is only possible if situational and task-specific influences are examined in addition to person-level determinants. Some seminal research has pursued this direction in the last two decades (e.g., Pychyl et al., 2000; Moon and Illingworth, 2005; Steel et al., 2018; van Eerde and Venus, 2018; Pollack and Herres, 2020). One of the findings of these studies is that an increased occurrence of procrastination behavior was related to everyday stresses (such as negative affect, Pollack and Herres, 2020; or poor sleep quality, van Eerde and Venus, 2018), providing additional support for the theoretical propositions of the mood-repair hypothesis (Sirois and Pychyl, 2013). The sophisticated experience sampling approach used in the present study helped to contribute and refine previous findings on the relationship between the affective experience of task aversiveness and the occurrence of task-specific delay behavior. Particularly noteworthy is the finding that the short-term increase in the appraisal of task aversiveness – at the moment when the intention was meant to be realized – significantly increased the risk for the occurrence of a delay. While we have not included any additional measures of students’ affective reactions to their tasks in the present study (besides asking about perceptions of aversiveness), we would like to advocate that future studies continue to test the assumptions of the mood-repair hypothesis (Sirois and Pychyl, 2013) by using very carefully planned sampling designs to contribute to a better understanding of the within-person processes that affect the occurrence of procrastination behavior under real-life conditions.

### The Impact of Between-Person Differences

The separate analyses revealed that delays were more likely to occur for students whose average task aversiveness appraisal in the initial intention formation exceeded the sample’s average by at least one standard deviation. This suggests that students who generally feel that their study-related tasks are highly aversive are more likely to delay working on their tasks. This finding corresponds with previous studies (e.g., Lay, 1992; Blunt and Pychyl, 2000; Ferrari and Scher, 2000; Pychyl et al., 2000). Under separate analysis, delays were significantly less likely to occur for students whose average expectations of success exceeded the sample’s average. Again, this is in line with previous research suggesting that students with stronger competency or self-efficacy beliefs are less likely to procrastinate than those who are less confident about their achievement potential (e.g., Wolters, 2003; Wäschle et al., 2014). However, there was no effect of students’ initial average evaluation for task aversiveness or their average expectation of success on the occurrence of behavioral delays in the combined analyses. Moreover, there was no effect of individual differences in students’ initial task-specific value appraisals on their delay behavior. Thus, our results do not suggest that some students are more likely to delay their study-related tasks because they have lower expectations of success in general. Likewise, it is not that behavioral delays become more or less likely because some students tend to assign higher personal value to their study-related tasks or experience all their tasks as more aversive than other students.

Instead, our results point to the fact that momentary within-person changes in the cognitive-affective appraisals of their tasks were the primary determinants of students’ delay behavior. In line with theoretical presumptions about the self-regulation of learning behavior (e.g., Winne and Hadwin, 1998; Boekaerts, 1999; Zimmerman, 2002; Pintrich, 2004), our findings suggest that students’ behavior is indeed strongly affected by the cognitive-affective appraisal of the task, which can change over time according to the prevailing situational or contextual conditions. It follows that the study of between-person differences should be complemented by studies clarifying more specifically which intra-individual (cognitive-affective) processes need to be effectively regulated by students in order to avoid delays in fulfilling their study-related tasks.

In the present study, individual differences in students’ self-reported general procrastination tendencies (measured by established questionnaires at baseline) did not predict their average risk for everyday dilatory behavior. In previous studies (e.g., Steel et al., 2001; Dewitte and Schouwenburg, 2002; Moon and Illingworth, 2005; Krause and Freund, 2014), procrastination tendencies measured by self-report questionnaires were weakly, or at best moderately correlated with observed behavioral delays (e.g., time until taking a test or handing in homework, or differences between the planned vs. actual time spent on learning activities). By showing that neither students’ general trait-based procrastination tendency nor their last week’s self-reported procrastination tendencies predicted their momentary task-specific procrastination behavior, our results are generally in line with findings suggesting that a merely trait-based explanation may not adequately describe the complex mechanisms involved in the occurrence of procrastination behavior (e.g., Steel et al., 2001; Moon and Illingworth, 2005). However, such a conclusion should be further substantiated by carefully designed studies and on the basis of a larger sample size (i.e., individuals at Level 2). In this connection, it should also be considered that the few longitudinal studies available have used distinct measures for both trait-procrastination and delay behavior, making it difficult to determine whether specific self-report questionnaires might be more or less suitable for predicting students’ actual delay behavior. We have therefore applied two different self-report questionnaires, one to assess students’ general procrastination tendency and one to assess students’ more proximal tendency to procrastinate on learning activities during the past week. Thus, it appears that it was not the different time- or content-specificity of the questionnaires used to capture students’ procrastination tendencies accounting for this lack of correlation with students’ self-reported actual delay behavior. Finally, it should be considered that the type of tasks students indicated in the e-diary may also have contributed to the fact that we found no strong effect of procrastination tendencies on the individual delay behavior reported in the e-diary. The short free-text inputs that students used to document their tasks were not very specific or elaborated. The entries consisted of single keywords (e.g., “complete worksheet,” or “tutorial”), which were visually checked for meaningfulness, but not qualitatively analyzed. This possibility would certainly be an informative endeavor that should be considered in future research.

### Implications for Research and Practice

The present work adds to an emerging effort to understand thewithin-person processes that affect students’ procrastinationbehavior over time and within their natural learning environment. Thepresent study cannot provide direct evidence for causality in therelationship between within-person changes in students’ task-specificappraisals and the occurrence of behavioral delays. However, ourstudy goes beyond the mere observation of delays in behavioraloutcomes and the examination of individual differences in students’ procrastination tendencies, ensuring that both the occurrence ofbehavioral delays and the behavioral determinants were captured bothin real-time and within real-life academic settings. The resultshighlight the importance of gaining a deeper insight into the dynamicprocesses that determine success or failure in students’ efforts torealize their task-specific intentions. It is reasonable to assumethat the detrimental changes in the task-specific appraisals that have been revealed in the present study may be more frequently experienced by more impulsive students who are less skilled in self-regulation (Steel, 2007), more intolerant toward the experience of negative emotions (e.g., Harrington, 2005), or less skilled in regulating the experience of negative emotions (e.g., Rebetez et al., 2015; Eckert et al., 2016). However, provided that procrastination behavior has been explained to arise because the avoidance of the negative affective experience that arises when dealing with a task that is perceived as aversive is prioritized over the benefit of long-term goal pursuit (Sirois and Pychyl, 2013), it is imperative to examine the momentary within-person processes that are involved. It is therefore essential that future studies continue and intensify previous efforts to use the far-reaching possibilities of intensive longitudinal assessments to gain a better understanding of the within-person processes that determine the success or failure of students’ self-regulatory efforts and influence their actual behavior in everyday academic life.

Knowledge of these processes will also benefit the development oflearning environments that support self-regulated learning processes. Students who procrastinate frequently will certainly benefit frommany of the already existing cognitive-behavioral interventions (see e.g., Schouwenburg et al., 2004; van Eerde and Klingsieck, 2018). However, the present findings suggest that approaches and interventions can be helpful, focusing less on changing the students than changing the instructional context and the tasks assigned to students. First, our result suggest that it can be helpful to strengthen students’ commitment to their study-related tasks and to support them to perceive their academic tasks as personally valuable (or relevant). Teachers should emphasize what students should learn by the tasks and how they can use this knowledge in later fields of application. This also entails setting tasks of practical relevance. Second, in order to support students’ expectations of success, it might be helpful if teachers express task requirements more explicitly, state what is expected, and by which criteria students’ performance is assessed. The importance of strengthening students’ efficacy expectations was also emphasized in previous research (e.g., Wolters, 2003; Wäschle et al., 2014). Wäschle et al. (2014) demonstrating that higher perceived self-efficacy to master study-related tasks protects against the occurrence of procrastination behavior and can be further enhanced by the experience of success. Setting adequate learning goals could contribute not only to strengthening students’ expectations of being effective in achieving their tasks but also to minimize their risk for behavioral delay (cf. Wolters, 2003; Wäschle et al., 2014).

Furthermore, the present findings emphasize the relevance of being equipped with effective self-regulation strategies and to apply them to work against the devaluation of a task. It has been previously demonstrated that trainings focusing on the ability to tolerate negative emotions can prevent the frequent occurrence of procrastination behavior (cf. Eckert et al., 2016). The finding that an increase in the aversiveness of a task at the moment when the intention to act was to be realized increases the risk to procrastinate further stresses the importance for students (at least for those with pronounced procrastination tendencies) to be trained in dealing with the experience of negative emotions. At the same time, it remains to be further clarified what contributes to students perceiving their academic tasks to be particularly aversive. Following Pekrun’s control-value theory of achievement emotions (Pekrun, 2006; Pekrun et al., 2007), a spontaneous increase in the perceived task aversiveness could indicate both students’ feeling bored in dealing with the task or that they feel overwhelmed. Both would suggest that moderately challenging tasks tailored to students’ abilities could reduce the risk for behavioral delays. Although this is a proposal to be backed up by future research, it is in line with Ferrari and Scher’s (2000) conclusion that tasks should be challenging but still enjoyable to increase the likelihood that students will perform them.

### Limitations

Some limitations of the present study should be taken into account, as they can also provide helpful information for future investigations. The first limitation refers to the possibility that our results may have been influenced by students’ reactivity to the e-diary. We tried to minimize potential biases due to social desirability effects by not explicitly asking students about their “procrastination” in the e-diary, but it is well possible that using the e-diary to evaluate their tasks and report their behavior regularly increased self-reflection (e.g., Barta et al., 2012; Conner and Reid, 2012) and thereby reduced the number of tasks that have been delayed. Moreover, the event-based experience sampling approach might itself serve as an intervention affecting the results. The instruction to state intentions about when one would like to work on a certain task the next day is similar to setting implementation intentions (Gollwitzer, 1999), which can be an effective strategy to prevent procrastination (e.g., Gollwitzer and Sheeran, 2006; Wieber and Gollwitzer, 2010), although there is also evidence that implementation intentions alone may be insufficient to prevent the occurrence of procrastination behavior or the occurrence of intention-behavior gaps (e.g., Cooke and Sheeran, 2004; Gustavson and Miyake, 2017). Moreover, the short free-text inputs that students used to document their tasks were not very specific or elaborated. The entries consisted of single keywords. Finally, if students would have delayed more of their tasks without using the e-diary, we might have even underestimated the effects of students’ task-specific appraisals on their delay behavior in the present study.

The second limitation refers to potential selectivity effects. Students participating in the present study were enrolled in cross-curricular courses advertised to help students self-organize their learning. We cannot rule out the possibility that self-selectivity effects may have affected the results, although the data analyzed to answer our research questions have been captured before any time-management or self-regulation strategies have been addressed. It is possible that students enrolled in the courses were highly motivated to change their behavior and have therefore procrastinated less, which should be considered as a major limitation when interpreting the results of our study. Although a comparison of our sample’s average scores with other (representative) student samples (e.g., Stöber, 1995; Helmke and Schrader, 2000) has shown that these were comparable in their average procrastination tendencies, it would be desirable to replicate our findings in a more representative student sample. It should also be mentioned that the large proportion of male students in our sample was most likely due to the fact that the proportion of male students in more technically oriented disciplines is still larger than in other disciplines. Thus, the results of our study may not be representative for students of all disciplines, as for example, there is usually a higher proportion of female students enrolled in the humanities or social sciences. Finally, students may also have been selective in the choice of the task-specific intentions they indicated. We cannot rule out the possibility that selectivity effects occurred due to the tasks that students have chosen (sampling of events analyzed at Level 1). Future studies could control objective features of the learning goals, ensuring that all students have to fulfill the same task (e.g., studying for the same exam) but set their own proximal learning goals.

Fourth, it should be noted that the sample size analyzed was relatively small to uncover individual differences in students’ procrastination behavior. According to the simulation study by Schoeneberger (2016), the sample size was sufficiently large to address our primary research question and provide reliable results. However, it cannot be ruled out that the finding that between-person differences in procrastination tendencies (captured by questionnaire measures) were not significantly related to individuals’ risk for delaying their tasks was a type two error. This finding should therefore be re-examined in studies with larger samples.

Fifth, the items used in the e-diary (presented in English andGerman in the Appendix) have not completely covered all facets of therespective constructs. The selection of items was theoreticallyjustified, but it was necessary to keep the number of items as small as possible when implementing the study. It can be assumed, for example, that the wording of the item used to measure students’ task value appraisals might not properly differentiate between the relevant components of “personal importance” and “attainment value” (cf. Trautwein et al., 2012; Dietrich et al., 2019). To address this limitation appropriately, future studies using a similar design should therefore focus on capturing individual appraisal dimensions in more detail.

Sixth, we want to mention the limitation that we cannot say whatcaused the within-person changes in students’ task-specificappraisals predicting behavioral delay. We do not know whether somedefensive mechanism caused the devaluation of a task (e.g., Knaus, 2000; Tuckman, 2005) to protect the self from the harmful recognition that one failed to follow one’s intention. It could be equally true that situational circumstances cause the devaluation. Future studies are needed to understand how task-specific cost-benefit considerations (cf. Flake et al., 2015) influence students’ decisions to learn (or work on their tasks) as intended or delay learning by engaging in alternative activities. It might also be informative in future studies to investigate whether the effects that were found in the present study differ between tasks that are study-related and those tasks from other (non-study-related) areas of life that have not been further examined in the present study.

## Conclusion

The present study examined the link between behavioral delays ingoal-directed actions by focusing on momentary within-person changes in students’ task-specific appraisals that may indicate a failure of self-regulation. Our findings support the view that the occurrence of delay behavior can be explained (in part) by within-person changes in cognitive-affective appraisals of tasks that appear between critical phases of goal pursuit. In contrast, students’ average risk to delay working on study-related tasks was not predicted by their general procrastination tendencies in the present study. These findings call for taking new perspectives in both research and teaching. More attention should be paid to the fact that students’ procrastination and learning behavior are determined by more than trait-based influences, attitudes, or abilities, but also by their perception of the task at hand and their affective experiences, which will both be considerably influenced by the context or situation. On the one hand, it is up to educators to ensure that students perceive the tasks assigned to them as a positive challenge, the accomplishment of which has practical, and thus personal, relevance. On the other hand, students will profit from trainings that strengthen their ability to effectively regulate their emotional reactions when dealing with aversive tasks. Finally, research must continue and increase the efforts to understand the (within-person) mechanisms that invoke self-regulated learning to fail and ultimately provoke students to delay working on their study-related tasks.

## Data Availability Statement

The raw data supporting the conclusion of this article will be made available by the authors, without undue reservation.

## Ethics Statement

Ethical review and approval was not required for the study on human participants in accordance with the local legislation and institutional requirements. The patients/participants provided their written informed consent to participate in this study.

## Author Contributions

LW was involved in conceptualization, data curation, formal analysis, investigation, methodology, project administration, software, validation, visualization, and writing – original draft. UE-P was involved in funding acquisition, project administration, resources, supervision, and writing – review and editing. ML was involved in data curation, project administration, software, and validation. UN was involved in funding acquisition, project administration, resources, supervision, and writing – review and editing. All authors contributed to the article and approved the submitted version.

## Conflict of Interest

The authors declare that the research was conducted in the absence of any commercial or financial relationships that could be construed as a potential conflict of interest.

## Footnotes

1 Most of the participants studied Architecture or Constructional Engineering (22.97%), Mathematics or Informatics (22.97%), Mechanical Engineering, Chemistry and Biosciences (17.57%), followed by Industrial Engineering and Economics (12.16%), Physics (9.46%), Arts and Humanities (8.11%), or Electrical Engineering and Information Technology (6.76%).

2 The study protocol included another second phase of experience sampling within the week of the third and final course session, finalized by (post-intervention) questionnaires. However, baseline assessments of procrastination tendencies and experience sampling data collected during the first eight days after the introductory session (i.e., before the intervention) were of exclusive relevance for answering the current research questions. We therefore focus on the first part of the study protocol for the remainder of the article.

3 The T1 measurement terminated when participants indicated that they would work on a task. When participants delayed their goal-directed action, they could decide to reschedule the event (i.e., enter a new trigger-time for the task, to begin later that day), or to delete the task from their daily schedule. Information collected during a third assessment unit (T2) – after a task was declared as completed – was not relevant for the current research question, and will not be further described in the present article.

4 We would like to thank a very attentive reviewer for drawing our attention to the fact that the proportion of tasks that have been delayed may seem relatively low with about 20%. In fact, we also expected a somewhat larger number of delays. While possible reasons for the low number of reported delays will be discussed in the limitations section, we would like to refer to a recently published experience-sampling study that reported a similarly low proportion of delayed learning sessions (i.e., 26%) among students studying for an exam (Gadosey et al., 2021).

5 Mplus indicates thresholds (instead of intercepts) for logistic regression analyses. The threshold reflects the value (the probability expressed in the logit of odds) that must be reached or exceeded to observe the event.

6 The Intraclass Correlation Coefficient (ICC) for a binary dependent variable can be computed using the formula ICC = $\sigma_{u}^{2}$/$\sigma_{u}^{2}$ + (π^2^/3), with $\sigma_{u}^{2}$ being the random intercept variance. As the Level 1 residual variance cannot be freely estimated; it is implicitly fixed to the standard logistic distribution variance π^2^/3 (cf. Schoeneberger, 2016; Sommet and Morselli, 2017).

7 The meaning of the predictor variables being zero depends on standardization and centering. The change index is zero when there is no change in the appraisal from T0 to T1. The assessment at T0 equals zero for a participants’ average evaluation of task value at Level 1 (due to person-mean centering). The Level 2 covariate (person mean) equals zero at the grand mean for the respective appraisal dimension. This principle applies to all models.

8 Regression coefficients (beta estimates) resulting from logistic regression represent the effect of the predictor on the log odds of the outcome [i.e., a task being delayed (*Y* = 1) vs. worked on (*Y* = 0) in a pairwise comparison] for a one-unit increase in the predictor variable. The odds ratio (OR) reflects the change in the likelihood that a task is delayed (*Y* = 1) vs. worked on (*Y* = 0) for each unit increase in the predictor. An OR < 1 indicates that the likelihood for the task being delayed is reduced when the predictor increases one unit. An OR > 1 indicates that the likelihood of the task being delayed increases when the predictor increases one unit.

9 It should be noted that Pekrun (2006) theory is generally in line with the basic proposition of appraisal theories, that the appraisal of the situation informs or defines the individual’s emotional response (cf. Frijda et al., 1989; Lazarus, 1993; Ellsworth and Scherer, 2003; Gross, 2015).

## Appendix

The e-diary items used in this study were presented to the students in German language and have been translated into English for the purpose of presentation in this publication. Below, we list the items used to assess students’ task appraisals in the original German wording and in the English translation. Each time, students were asked to indicate their appraisals, the respective task appeared on the screen, followed by the items:

**Table:**

Items used to assess students’ task appraisal in the planning phase (T0).		
**Appraisal**	**German wording**	**English translation**
Value	Wie wichtig ist es Ihnen persönlich, dass Sie diese Aufgabe morgen erledigen/dieses Tagesziel erreichen?	How important is it to you personally that you work on that task/reach that goal tomorrow?
Expectation	Wie schätzen Sie die Wahrscheinlichkeit ein, dass Sie diese Aufgabe morgen tatsächlich erledigen/dieses Tagesziel tatsächlich erreichen?	How likely do you think it is that you will work on that task/reach that goal tomorrow?
Aversiveness	Wie (un-)angenehm ist diese Aufgabe/die Bearbeitung dieses Tagesziels für Sie?	How (un-)pleasant is this task/working on this goal?
Effort	Wie viel Anstrengung müssen Sie voraussichtlich investieren um diese Aufgabe zu bearbeiten/dieses Tagesziel zu erreichen?	Prospectively, how much effort do you have to invest to work on this task/reach this goal?

**Items used to assess students’ task appraisal in the implementation phase (T1).**		

**Appraisal**	**German wording**	**English translation**

Value	Wie wichtig ist es Ihnen persönlich, dass Sie diese Aufgabe jetzt erledigen/dieses Tagesziel erreichen?	How important is it to you personally that you work on that task/reach that goal right now?
Aversiveness	Wie (un-)angenehm ist diese Aufgabe/die Bearbeitung dieses Tagesziels jetzt gerade für Sie?	How (un-)pleasant is this task/working on this goal right now?
Effort	Wie viel Anstrengung müssen Sie jetzt investieren um diese Aufgabe zu bearbeiten/dieses Tagesziel zu erreichen?	How much effort do you have to invest right now to work on this task/reach this goal?
